# Anxiogenic Potential of Experimental Sleep Fragmentation Is Duration-Dependent and Mediated via Oxidative Stress State

**DOI:** 10.1155/2021/2262913

**Published:** 2021-08-21

**Authors:** Željko Grubač, Nikola Šutulović, Sonja Šuvakov, Djurdja Jerotić, Nela Puškaš, Djuro Macut, Aleksandra Rašić-Marković, Tatjana Simić, Olivera Stanojlović, Dragan Hrnčić

**Affiliations:** ^1^Institute of Medical Physiology “Richard Burian”, Belgrade University Faculty of Medicine, 11000 Belgrade, Serbia; ^2^Institute of Clinical and Medical Biochemistry, Belgrade University Faculty of Medicine, 11000 Belgrade, Serbia; ^3^Institute of Histology and Embryology “Aleksandar Đ. Kostić”, Belgrade University Faculty of Medicine, 11000 Belgrade, Serbia; ^4^Clinic of Endocrinology, Diabetes and Metabolic Disease, UCCS, Belgrade University Faculty of Medicine, 11000 Belgrade, Serbia

## Abstract

Sleep architecture alterations, among which sleep fragmentation is highly prevalent, represent risk factors for a variety of diseases, ranging from cardiovascular to brain disorders, including anxiety. What mediates anxiety occurrence upon sleep fragmentation is still a matter of debate. We hypothesized that the sleep fragmentation effects on anxiety are dependent on its duration and mediated by increased oxidative stress and alterations in the number of parvalbumin (PV+) interneurons in the hippocampus. Sleep was fragmented in rats by the treadmill method during a period of 14 days (SF group). Rats with undisturbed sleep in the treadmill (TC group) and those receiving equal amounts of treadmill belt motion (EC group) served as controls. To assess anxiety, we subjected rats to the open field, elevated plus maze, and light-dark tests on the 0, 7^th^, and 14^th^ day. Upon the last test, brain structures were sampled for oxidative stress assessment and PV+ interneuron immunohistochemistry. The results of ethological tests of anxiety-linked behavior suggested duration-dependent anxiogenic potential of sleep fragmentation. Rats' anxiety-linked behavior upon sleep fragmentation significantly correlated with oxidative stress. The rats with fragmented sleep (SF) showed significantly higher oxidative stress in the hippocampus, thalamus, and cortex, compared to controls (TC and EC), while the antioxidant enzymes' activity was significantly decreased. No significant differences were observed in hippocampal PV+ interneurons among these groups. Our results showed that duration of sleep fragmentation is a significant determinant of anxiety-linked behavior, and these effects are mediated through oxidative distress in the brain. Herein, it is revealed that the sleep fragmentation-oxidative stress-anxiety axis contributes to our better understanding of pathophysiological processes, occurring due to disrupted sleep patterns.

## 1. Introduction

Sleep is a vital physiological process widely preserved during phylogenies, indicating its utmost importance for the functioning of the whole organism. Nowadays, sleep disorders are widely prevalent in the general population and include alterations in quantity and quality of sleep [1]. Different etiological factors, ranging from lifestyle and environmental factors to specific medical entities, such as obstructive sleep apnea (OSA), may provoke sleep continuity disruption, i.e., fragmentation of sleep. In contrast to total sleep deprivation, sleep fragmentation is the set of frequent arousals, restructuring the basic sleeping architecture, rather than the loss of total sleep time [2, 3]. Clinically, it appears as daytime somnolence, and it adversely affects mood, cognition, and memory [4, 5]. Any variations in the sleep architecture, especially when it is prolonged, can cause serious medical problems, since sleep plays a pivotal role in brain homeostasis [6, 7]. Namely, it can be a risk factor for panic attacks, manic episodes [8], paranoid schizophrenic-like behavior [9], and other conditions with anxiety in the intersection.

Sleep fragmentation (SF) is one of the essential features of OSA, concomitantly occurring in these patients with intermittent hypoxia [10]. OSA patients may arouse up to several hundred times per night, leading to a loss of sleep quality [11]. Anxiety is highly prevalent in patients with OSA with an increase in self-reported anxiety, somnolence [12, 13], and a higher level of traffic accidents [14–16]. Majority of studies on anxiety in apnea patients focused primarily on intermittent hypercapnia [17] as a possible underlying mechanism, which left an open question of how sleep fragmentation in OSA contributes to psychiatric comorbidities. However, sleep and anxiety are closely related [18]. Complaints such as insomnia or nightmares have even been incorporated in some anxiety disorder definitions [19–21]. Therefore, the role of sleep fragmentation in the mediation of anxiety in OSA patients seems to be very rational to hypothesize. Indeed, the models of acute sleep fragmentation recently developed in rodents confirmed an effect on cognition, in particular, memory consolidation, brain excitability, and anxiety-related behaviors [22–25]. However, we still do not have clear experimental data on the relationship between sleep fragmentation in OSA and anxiety-linked behavior, nor the underlying molecular mechanisms.

The presence of oxidative stress, a state when reactive oxygen species (ROS) concentration exceeds the antioxidative capacity of an organism [26], has already been demonstrated in patients with OSA. However, these studies also focused on intermittent hypoxia and hypercapnia, showing improvement upon continuous positive airway pressure therapy [27, 28]. On the other hand, the connection between impaired sleep quality and oxidative damage is still controversial, and there is evidence of pros and cons for the idea of this type of interaction [29]. It is also evident that oxidative stress could play a role in the pathophysiology of anxiety [30–32] showing existence of increased oxidative species and decrease in antioxidative defense in animals and patients with anxiety, as well as the beneficial effects of anxiolytics on the oxidative stress level [33–35].

Recently, it has been shown that parvalbumin-positive (PV+) cells could be particularly vulnerable to oxidative stress induced by sleep deprivation, showing their possible involvement in different outcomes of sleep modulation. On the other hand, PV+ interneurons are highly represented in the hippocampus [14], and their dysfunction has been demonstrated in various psychiatric illnesses, including generalized anxiety disorder [16].

Our study is aimed at determining changes in mental (anxiety-like behavior) and neurochemical (the brain oxidative status) status after chronic sleep fragmentation, together with a number of hippocampal PV+ interneurons (a marker of mood disorders). We hypothesized that prolonged sleep fragmentation in rodents is involved in anxiety-like behavior with increased oxidative stress damage and decreased number of hippocampal PV+ interneurons.

## 2. Materials and Methods

### 2.1. Animals

Experiments were performed on adult males of *Wistar albino* rats. They were two-month-old and weighted 180–200 g at the beginning of the study. Rats were obtained from a local certified breeding facility and had been acclimatized to our laboratory conditions (temperature range of 22-24°C, relative humidity range of 50 ± 5%, with 12 h long light period from 8 a.m. to 8 p.m.) for 7 days. Access to food and water was *ad libitum*. They were kept under controlled ambient conditions. Animals were used only once during the experiment.

### 2.2. Ethical Disclosure

Our study design, number of animals, and procedures were approved by National Animal Care authorities (Permission 323-07-05290/2017-05). We hold to the Directive of the European Parliament and the Council (2010/63/EU) in all experimental procedures.

### 2.3. Experimental Design and Chronic Sleep Fragmentation

#### 2.3.1. Experimental Design

**Figure 1:**
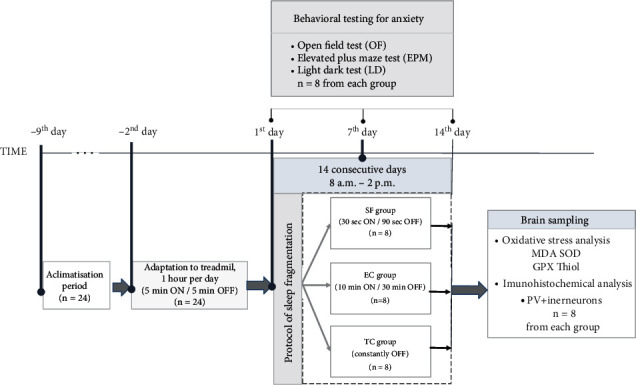
Experimental design graphical outline. Acclimatization to laboratory conditions was allowed immediately upon the animal arrival for 7 consecutive days (-9^th^ to -2^nd^ days). Sleep was fragmented in rats by treadmill method during 14 consecutive days (SF group). Rats with undisturbed sleep in treadmill (TC group) and those receiving equal amount of treadmill belt motion (exercise, EC group) served as controls. Behavioral testing for anxiety was performed on day 0 (baseline), as well as on the 7^th^ and 14^th^ days upon protocol initiation. On the 14^th^ day, after the experiments were finished, all animals were sacrificed for brain structure isolation. One of the brain hemispheres (alternately left or right) was used for brain oxidative stress assessment, while another one is processed for immunohistochemical analysis of the hippocampal PV+ interneuron number.

The study design graphical outline is presented in Figure 1. Upon acclimatization to the laboratory conditions, the animals were adapted to treadmill apparatus for small laboratory animals, used in the herein applied protocol of sleep fragmentation, according to accepted methodology described in detail previously [27–29]. Sleep was fragmented in rats by the treadmill method during 14 consecutive days (SF group). The rats with undisturbed sleep-in treadmill (TC group) and those receiving an equal amount of treadmill belt motion (exercise, EC group) served as controls. We subjected the rats from these groups (*n* = 8 per group) to ethological assessment tests of anxiety-linked behavior at different time points during the experiment, i.e., on day 0, day 7, and day 14 of the sleep fragmentation protocol. The standard battery of these tests consisted of an open field, light/dark, and elevated plus maze tests to which the rats were subjected, respectively. On day 14, the rats were sacrificed for brain structure isolation. One of the brain hemispheres (alternately left or right) was used for brain oxidative stress assessment, while the other one is processed for immunohistochemical analysis of the hippocampal PV+ interneuron number.

#### 2.3.2. Protocol of Sleep Fragmentation

We used an established methodology, described in detail previously, to fragment sleep in rats during the first 6 hours of the light phase of the light/dark cycle. Briefly, treadmill activity was defined by the ON mode (working mode, belt moving) at the speed of 0.02 m/s and OFF mode (stop mode) at the speed of 0 m/s. The treadmill was programmed to work alternately for the 30 s ON and 90 s OFF every 2 minutes during the entire period of 6 h to achieve sleep fragmentation in the SF group. The applied frequency of fragmentation is similar to that in patients with OSA. In order to control any confounding effects of the belt drive, the corresponding activity control group was also formed. In this group, the total movement that rats received was equal to the SF group but without disruption of their sleep during larger periods of time (10 min ON and 30 min OFF, exercise control group, EC). Finally, the treadmill control (TC) group consisted of rats staying in the treadmill apparatus without belt movement, with conditions equivalent to those in their home cages. After each treadmill session, the rats within the SF, EC, and TC groups were transferred to home cages, and the procedure was repeated in the same manner the next day for fourteen consecutive days.

### 2.4. Tests of Anxiety-Like Behavior

#### 2.4.1. Open Field Test

We used the classical open field test, as described in detail previously [32]. In this set-up, rat behavior in the open field arena was monitored by infrared sensors (Experimetria Ltd., Budapest, Hungary) and analyzed offline by dedicated software (Conducta 1.0). During rat exploration of the novel environment in the session of 15 min, the system recorded horizontal activity parameters: the distance and time of ambulatory movement, as well as the vertical activity parameters: number of rearings. For the purposes of anxiety-linked behavior analysis, the entire open area was divided into 16 square fields, of which the 4 middle square fields were denoted as the central area. The system also measured the time that an animal spent in the central area. The thigmotaxic index was calculated as a ratio between the distance of rat ambulation in the peripheral zones and the total ambulation distance, expressed in %.

#### 2.4.2. Light-Dark Test

The light-dark test consisted of the two compartments interconnected by a square aperture: the light compartment with all sides dyed in white and the dark compartment with all sides dyed in black (Elunit, Belgrade, Serbia). The test begins with the rat being gently placed in the center of the light compartment, and it ends 5 minutes later. Activity of the rat was video recorded and analyzed offline by an investigator blinded to the treatment.

The parameters of anxiety-linked behavior derived from this test were (i) the time animal spent in the light compartment and (ii) the number of transitions from the light to the dark compartment.

#### 2.4.3. Elevated Plus Maze Test

In the apparatus of the elevated plus maze test, the pair of open arms crossed with the pair of enclosed arms in the position of the central platform (Elunit, Belgrade, Serbia). Crossing arms were raised to a height of 50 cm above the floor level. The test begins with the rat being gently placed on the central platform, facing an open arm, and it ends 5 min later. Activity of the rat was video recorded and analyzed offline by an investigator blinded to the treatment.

The parameters of anxiety-linked behavior derived from this test were (i) the time animal spent in the open arms and (ii) the number of open-closed arm transitions.

### 2.5. Biochemical Analysis of Oxidative Stress

As described in the study design, brain samples were collected upon completing all behavioral tests. The hippocampus and cortex were carefully dissected. Tissue samples were homogenized in RIPA buffer (8 volumes). Protease inhibitor cocktail was also added. The supernatant was collected after the tubes were centrifuged for 30 min (14,000 rpm at 4°C). The following parameters were measured: (i) malondialdehyde (MDA) concentration, (ii) superoxide dismutase (SOD) activity, (iii) glutathione peroxidase (GPx) activity, and (iv) protein thiol (P-SH) groups.

MDA concentration was determined by the colorimetric method of Dousset et al. [36]. MDA conjugates with thiobarbituric acid reactive substances (TBARS) and forms an MDA-TBA complex of red color with its light absorption peak at 532 nm and molar absorption coefficient of 1.56 × 10^5^ L/(mol × cm).

SOD activity was determined by Misra and Fridovich [37]. SOD inhibits autooxidation of epinephrine at alkaline pH (10.2); hence, the SOD activity unit was expressed as the amount of enzyme necessary to inhibit the oxidation of epinephrine by half.

GPx activity was determined by the coupled assay procedure [38]. GPx activity unit was expressed as mmol NADPH oxidized/min. We assumed 6.22 × 10^3^/L/mol/cm to be the molar absorbency of NADPH at 340 nm.

P-SH were determined by the method of Jocelyn [39]. P-SH reduces DTNB (5,5-dithiobis-(2-nitrobenzoic acid)) making 5-thio-2-nitrobenzoic acid (TNB) of yellow color with a molar extinction coefficient of 13.6 × 10^3^ L mL/1 cm at 412 nm wavelength.

### 2.6. Hippocampal PV+ Neuron Determination by Immunohistochemistry

Brain hemispheres (alternately left or right) isolated from rats of TC, EC, and SF groups were embedded in paraffin upon fixation (4% formaldehyde in PBS). For antigen retrieval, we used 5 *μ*m thick, dewaxed, and rehydrated coronal brain sections which were treated with citrate buffer (pH 6.0) in a microwave. Normal horse serum was used to block nonspecific labeling. Endogenous peroxidase activity was blocked with 3% H_2_O_2_. Slices were incubated in primary antibody-mouse monoclonal anti-PV (1 : 1000, Sigma-Aldrich) overnight at room temperature. A biotinylated anti-mouse secondary antibody followed by an avidin-biotin-horseradish peroxidase complex (Vector Laboratories) was used for labeling. The 3,3′-diaminobenzidine chromogens were used for visualization of the immunoreactive sites (Vector Laboratories). Mayer's hematoxylin was used to counterstain the sections. A Leica DM4000 B LED microscope with digital camera Leica DFC295 using the Leica Application Suite (LAS, v4.4.0) software system was used for counting. Counting of the immune-reactive neurons was done on the dorsal hippocampus and its regions: CA1, CA2/3, and dentate gyrus (DG). We have chosen to evaluate changes in the hippocampus, a part of the limbic system with a pivotal role in anxiety pathogenesis, since it has been shown that behavioral alterations, including anxiety, were coupled with alterations in the number of dark neurons and diameter of the C region of the hippocampus and an increase in the percent of dark neurons as well as a decrease in diameter of the CA3 area of the hippocampus [30, 40].

The number of immune-reactive neurons is expressed per 1 mm^2^ of the investigated hippocampal region. Counting was done by two independent investigators blinded to the group allocation. The mean value was taken as the final count, since high (Pearson's *r* = 0.95) interobserver reliability was obtained.

### 2.7. Data Analysis

The Shapiro-Wilk test was used to test the normal distribution of values. The analyzed parameters obtained from the open field, light/dark, and elevated plus maze test, as well as the oxidative stress values and number of PV+ interneurons, showed normal distribution. For these parameters, the results were expressed as a means ± standard error (SE). The statistical difference between the groups was estimated by using one-way ANOVA with Fisher's LSD post hoc test. The exceptions were the number of transitions in the light/dark test and elevated plus maze, for which the normal distribution was not estimated. Results on these parameters were expressed as medians with 25^th^–75^th^ percentiles, and statistical difference between the groups was estimated by Kruskal-Wallis one-way ANOVA with Mann–Whitney *U* test. Statistical significance of within-group differences for output variables was assessed by ANOVA for repeated measurements. The significance of statistical differences was set up as *p* < 0.05 and *p* < 0.01.

## 3. Results

### 3.1. Anxiety-Like Behavior

#### 3.1.1. Open Field Test

**Figure 2:**
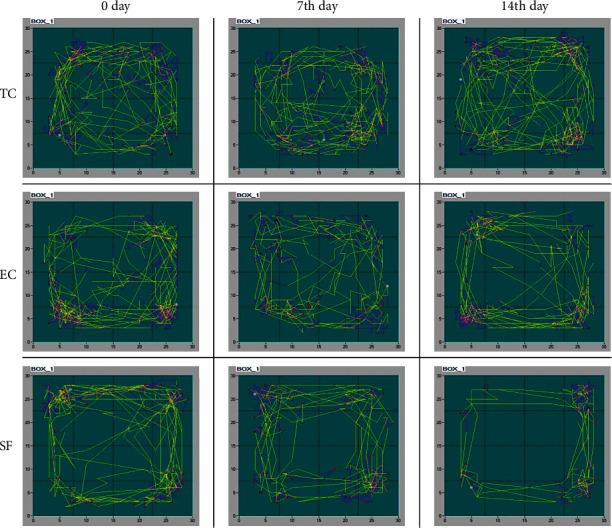
Representative traces of locomotor activity in the open field test. Representative traces of locomotor activity in the open field test of animals within the TC, AC, and SF groups at the beginning (day 0, baseline), as well as at 7^th^ and 14^th^ days upon sleep fragmentation protocol. For details, see Figure 1.

Representative traces of animals' locomotor activity within the TC, EC, and SF groups at the beginning (day 0) as well as at the 7^th^ and 14^th^ day upon the sleep fragmentation protocol are presented in Figure 2.

As it could be noted, behavioral patterns were visually similar in the TC and EC groups over the experimental period, but differences were apparent on the 7^th^ and 14^th^ days in the SF group.

**Figure 3:**
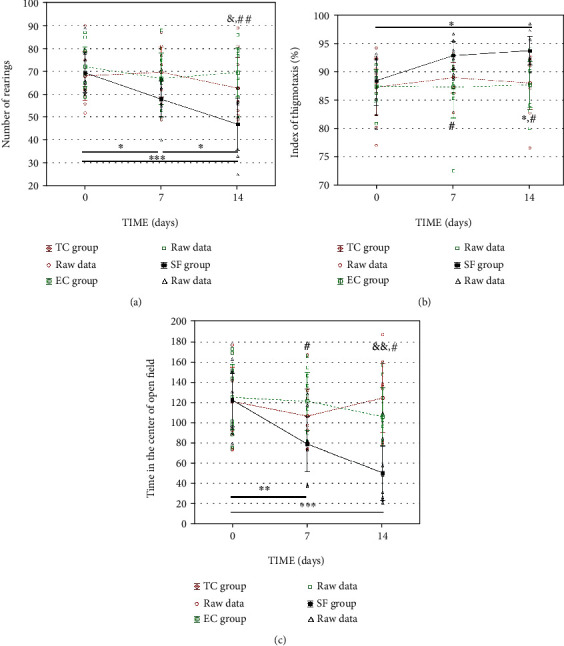
Quantitative evaluation of the anxiety-linked parameters in the open field test. The number of rearings (a), index of thigmotaxis (b), and time spent in the central area (c) in the open field test (OF) registered in the AC, EC, and SF groups. The index of thigmotaxis was calculated as a ratio between the distance of ambulatory movement in the peripheral zones and the total distance of ambulatory movements in the OF. Values are expressed as the mean ± SEM. Within-group differences were estimated by one-way ANOVA with the Tukey-Kramer LSD post hoc test. ^∗^*p* < 0.05, ^∗∗^*p* < 0.01, and ^∗∗∗^*p* < 0.001 within the SF group. The same tests were used to estimate the differences between the groups. ^#^*p* < 0.05 and ^##^*p* < 0.01 vs. TC; ^&^*p* < 0.05 and ^&&^*p* < 0.01 vs. EC. For details, see the caption of Figure 1.

Quantitative evaluation of the anxiety-linked parameters in the open field test showed no differences within the TC group over time in any of the investigated output parameters (0, 7, and 14 days, *p* > 0.5, Figure 3). The same holds for the parameters registered in the EC group. According to one-way ANOVA, differences were revealed in rats within the SF group over 7 and 14 days. The number of rearings, an indicator of vertical activity, was significantly decreased in SF rats compared to EC rats (*p* < 0.01), as well as to the TC group (*p* < 0.05). The number of rearings decreased gradually over time, i.e., it was significantly lower upon 14 days compared to the value observed upon 7 days (*p* < 0.05) (Figure 3(a)).

The index of thigmotaxis was significantly higher in SF rats, in comparison to rats from TC (*p* < 0.05), as well as from the EC (*p* < 0.05) group, at the end of the fragmentation period. Also, the index of thigmotaxis in SF rats 14 days upon fragmentation was significantly (*p* < 0.05) lower compared to their baseline values (Figure 3(b)).

**Figure 4:**
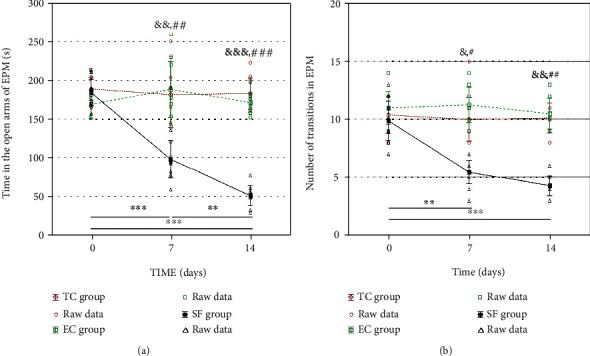
Behavioral patterns observed in the elevated plus maze test: the time spent in the open arm (a) and the number of transitions between the open and closed arms (b). Values are expressed as the mean ± SEM. Within-group differences were estimated by one-way ANOVA with Tukey-Kramer LSD post hoc test. ^∗∗^*p* < 0.01, ^∗∗∗^*p* < 0.001 within the SF group. The same tests were used to estimate the differences between the groups. ^#^*p* < 0.05, ^##^*p* < 0.01, and ^###^*p* < 0.001 vs. TC; ^&^*p* < 0.05, ^&&^*p* < 0.01, and ^&&&^*p* < 0.001 vs. EC. For details, see caption of Figure 1.

We also analyzed the time that the animals spent in the central area of the open field (Figure 3(c)). Rats from the SF group spent significantly less time in the central area compared to the rats from the TC and EC groups (*p* < 0.01, Figure 4) at the testing point of 14 days and compared to rats from the EC group at the testing point of 7 days (*p* < 0.05). Besides, there is a highly significant reduction of the time spent in the central area of the open field test, within the SF rats on the 14^th^ day in comparison to basal values (*p* < 0.001) and testing on the 7^th^ day (*p* < 0.01).

#### 3.1.2. Elevated Plus Maze Test

Results of the elevated plus maze tests are represented in Figure 4.

**Figure 5:**
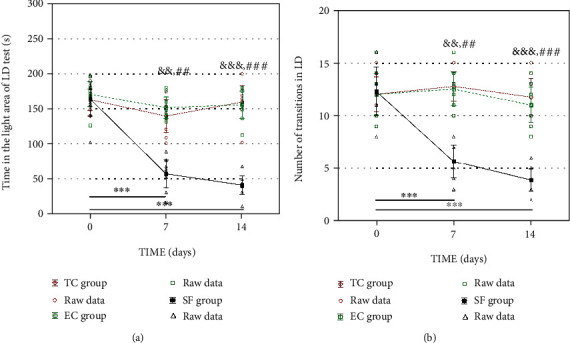
Behavioral patterns observed in the light-dark test: the time in the light compartment (a) and the number of transitions between the light and the dark compartments (b). Values are expressed as the mean ± SEM. Within the group, differences were estimated by one-way ANOVA with Tukey-Kramer LSD post hoc test. ^∗∗∗^*p* < 0.001 within the SF group. The same tests were used to estimate the differences between the groups. ^##^*p* < 0.01 and ^###^*p* < 0.001 vs. TC; ^&&^*p* < 0.01 and ^&&&^*p* < 0.001 vs. EC. For details, see caption of Figure 1.

No differences among the TC, EC, and SF groups in any of the analyzed parameters resulting from elevated plus maze test were detected at baseline measurement (0 day). On the other hand, sleep fragmentation protocol (SF group) significantly decreased the time animals spent in the open arms during the test compared to the TC group (SF *vs*. TC, Figure 5(a)) at 7 (*p* < 0.01) and 14 (*p* < 0.001) days of sleep fragmentation, as well as compared to the EC group (SF *vs*. TC, Figure 4(a)) on the 7^th^ (*p* < 0.01) and 14^th^ (*p* < 0.001) days. The time that SF rats spent in the open arms decreased gradually over time, i.e., it was significantly lower upon 14 days, compared to the value observed upon 7 days (*p* < 0.001), and 7 days compared to the baseline value (*p* < 0.001). The same holds true for differences observed for the number of open/closed arm transitions in these groups over 7 and 14 days, as presented in Figure 4(b).

#### 3.1.3. Light/Dark Test

Results derived from the light/dark test are presented in Figure 5.

None of the light/dark test parameters were different among the TC, EC, and SF groups at baseline measurement (*p* > 0.05). The time that the rat spent in the light compartment was significantly shorter in the SF group compared to the TC and EC groups on the 7^th^ (*p* < 0.01) and 14^th^ (*p* < 0.001, Figure 5(a)) day testing points. The same holds for the number of light/dark compartment transitions (Figure 5(b)).

### 3.2. Oxidative Stress in the Brain

**Figure 6:**
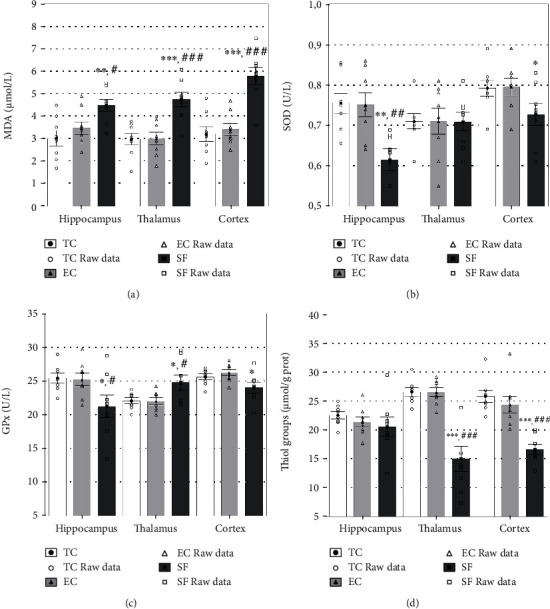
Oxidative stress in the hippocampus, thalamus, and cerebral cortex upon completion of sleep fragmentation. Malondialdehyde level (MDA, a), the activity of glutathione peroxidase (GPx, b), the activity of superoxide dismutase (SOD, c), and the level of thiol groups (d) determined in the brain structures in the TC, EC, and SF groups. Values are expressed as the mean ± SEM. ANOVA with Tukey-Kramer LSD post hoc test was used to estimate the differences between the groups. ^∗^*p* < 0.05, ^∗∗^*p* < 0.01, and ^∗∗∗^*p* < 0.001 vs. TC; ^#^*p* < 0.01, ^##^*p* < 0.01, and ^###^*p* < 0.001 vs. EC. For details, see Figure 1.

Sleep fragmentation affected significantly oxidative stress burden in the brain of the rats. Thus, MDA concentration was significantly higher in all three explored structures of the brain compared to the rats within both control groups (Figure 6(a)). No differences were observed between the TC and EC groups.

Sleep fragmentation also induced alterations in the activity of antioxidant enzymes in the examined brain structures. The activity of the GPx enzyme was significantly lower in the hippocampus, thalamus, and cortex of the rats within the SF group compared to rats within the TC group (*p* < 0.05, Figure 6(b)), as well as compared to the EC group in the hippocampus and thalamus (*p* < 0.05). No differences were observed between the TC and EC groups.

The SOD activity in sleep-fragmented animals was clearly lower in the hippocampus and cortex. While the SOD activity measured in the hippocampal structures was significantly decreased in the SF group compared to both TC and EC control groups, the SOD in the cortex differed only in the treadmill control group. Sleep fragmentation did not alter SOD activity in the thalamus (Figure 6(c)). The animals either from the TC or EC groups did not differ regarding SOD activity in any of the explored structures.

Protein oxidative damage was assessed as a total thiol content in the brain tissue lysates. While the sleep fragmentation did not significantly affect thiol content in the hippocampus (*p* < 0.05, Figure 6(d)), a significant loss was observed in the thalamus and cortex of sleep-fragmented rats, compared to both treadmill and activity control groups. No difference regarding thiol content was observed between the TC and EC groups.

### 3.3. Correlation Analysis

**Figure 7:**
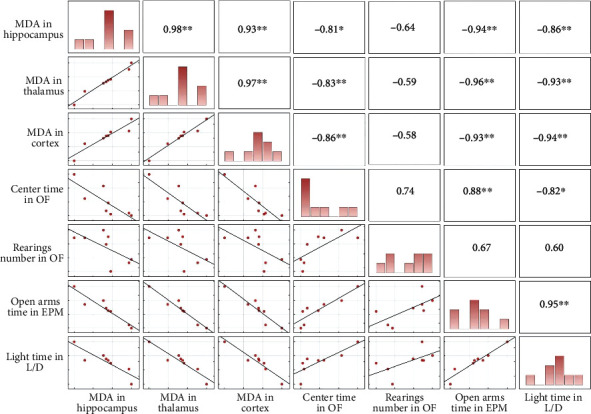
Correlation analysis between the MDA levels in the hippocampus, thalamus, and cortex with parameters of anxiety-linked behavior. Correlation matrix showing the correlation between the MDA levels in these three brain structures and output variables from behavioral tests. OF: time in the center; EPM: time in the open arms; LD: time spent in the light compartment. The distribution of each variable is plotted diagonally. The upper triangular matrix encompasses Pearson's *r*. The lower triangular matrix is composed of scatter plots showing the variable's relationships. ^∗^*p* < 0.05 and ^∗∗^*p* < 0.01. For details, see caption of Figure 1.

In order to evaluate the link between the hippocampus, thalamus, and cortex MDA level with anxiety-linked behavior, a Pearson correlation coefficient was calculated, and the results of the correlation matrix derived from this analysis are presented in Figure 7.

There were strong negative correlations between the MDA level in the hippocampus, thalamus, and cortex and the time SF rats spent in the center of the open field (*r* = −0.81, *p* < 0.05; *r* = −0.83, *p* < 0.01; and *r* = −0.86, *p* < 0.01). There was no statistically significant correlation between rearings in the open field and MDA level in any of the brain structures from SF rats.

There were strong negative correlations between the MDA level in the hippocampus, thalamus, and cortex and the time SF rats spent in the open arms of the EPM test (*r* = −0.94, *p* < 0.01; *r* = −0.96, *p* < 0.01; and *r* = −0.93, *p* < 0.01), as well as the time rats spent in the light compartment of the light/dark box (*r* = −0.86, *p* < 0.01; *r* = −0.93, *p* < 0.01; and *r* = −0.94, *p* < 0.01).

### 3.4. Hippocampal PV+ Interneuron Analysis

**Figure 8:**
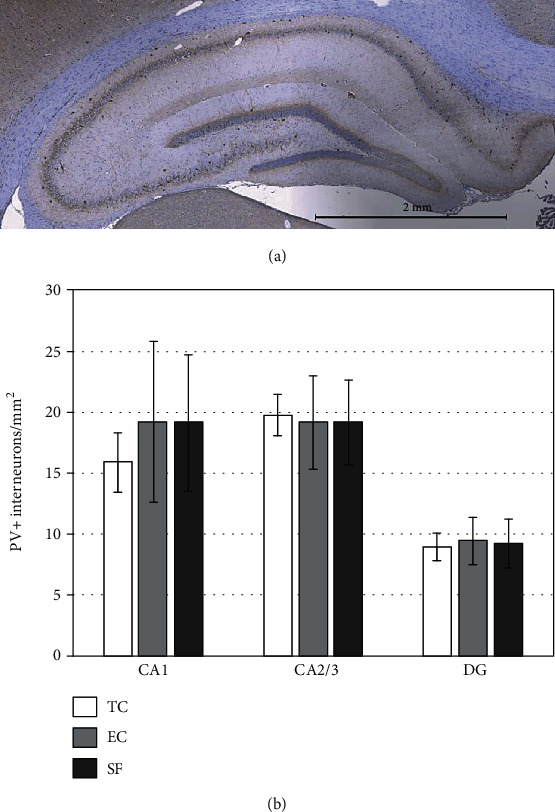
Hippocampal PV+ interneuron analysis. Hippocampal representative micrograph with denoted CA1, CA2/3, and DG regions (a). Scale: 1 mm. PV+ interneuron number in the hippocampal regions (b). Values are the mean ± SEM. ANOVA with Tukey-Kramer LSD post hoc test was used to estimate the differences between the groups. For details, see Figure 1.

Immunohistochemistry staining identified PV+ interneurons, mainly in the area of the pyramidal cell layer in CA1 and CA2/3 and commonly in the granular cell layer in DG (Figure 8). Quantification of PV+ interneuron numbers in these three hippocampal regions revealed no significant differences between the TC, EC, and SF groups of rats (*p* > 0.05).

## 4. Discussion

The results of the open field, elevated plus maze, and light-dark tests, ethological tests of anxiety-linked behavior, performed in this study suggested a duration-dependent anxiogenic potential of sleep fragmentation. Further biochemical analysis showed that the rats with fragmented sleep (SF) showed significantly higher oxidative stress in the hippocampus, thalamus, and cortex compared to controls (TC and EC), while the antioxidant enzymes' activity was significantly decreased. Correlation analysis showed that rat anxiety-linked behavior upon sleep fragmentation significantly and positively correlated with oxidative stress. On the other hand, results of immunohistochemical study showed that no significant differences were observed in hippocampal PV+ interneurons among these groups.

Previous results on the effect of disrupted sleep architecture on anxiety-linked behavior in rats seemed to be inconsistent, and the exact reasons are still being discussed [2, 41]. In this study, we used a model of prolonged sleep disruption of 6 hours per 24 hours for 14 days. In this type of setting, the REM rebound effect is possible to happen. However, it has been proven that it disappears after the first day [42]. We chose a schedule of 30 s of sleep disruption at 2 min intervals during the 6 h light span, the period in which most sleep occurs. This protocol is chosen to better mimic the changes in patients with severe sleep apnea, in which periods of hypopnea or apnea often last for 10–60 s at 1–2 min intervals. SF (6 h) was associated with transient increases in the proportion of time spent in slow-wave sleep SWS and REMs during some 2 h intervals within the 18 h period after SF as compared to the same interval on the baseline [43]. In a study conducted by Trammell et al. [43], mice exposed to 6-hour fragmentation completely recovered lost REM sleep during the subsequent 18 h period in which uninterrupted sleep was permitted yet did not recover lost SWS and retained a significant SWS debt at the end of the recording period. That was not the case with the 12 h/24 h or 24 h/24 h fragmentation, because an SWS debt was also maintained after a recovery period, equivalent to the period of SF, but without recovery in lost REM sleep [43]. This was also one of the reasons why we used 6-hour sleep fragmentation instead of 12 or 24 hours.

To our knowledge, this is the only study that included both subacute and chronic effects of disrupted sleep quality on anxiety in the same group of animals. The results of open field testing showed a reduction of the interest in exploring the unknown environment and decreased the number of rearing. It also showed a significant increase in the thigmotaxic index in the SF group. The rearing number reduction also gives us the image of reduced exploration interest. A behavioral pattern characterized by an obvious decline in time spent in the central area and rises in the thigmotaxis index, along with a decline in the number of rearings on the 14^th^ day compared to the 7^th^ day in the SF group, clearly shows the impact that duration of fragmented sleep has. The results of both the light/dark test and elevated plus maze test are in line with the results of the open field test, being also more severe in chronic (14 days) compared to subacute (7 days) conditions. To sum up, behavioral results of all three tests are suggesting a strong, duration-dependent, anxiogenic potential of sleep fragmentation.

Although OSA patients are believed to have poor mental health [44, 45], some studies failed to show a significant correlation between OSA and psychiatric disease [46]. Some researchers hypothesized that the cause of this connection could be found in sleep fragmentation or poor sleep quality [41, 47]. Herein, high-frequency chronic sleep interruption expressed the potential to increase anxiety-like behavior in male rodents, which is in agreement with results of Sateia [48] who showed that disturbance of sleep quality and continuity predisposes to the development or exacerbation of mental illness [48]. On the contrary, our results refute the theory that psychopathology in OSA may represent both an etiological factor and a complication [49] since we clearly showed that sleep disruption, similar to one in sleep apnea, causes anxiety in experimental conditions. Once developed, anxiety could further worsen the sleep quality, therefore creating a loop, as sleep impairment and mental health may originate from the same neurobiological processes [49], which is probably the case here.

Oxidative stress, as a potential mechanistic basis of anxiety, developed due to sleep restriction/fragmentation, has not been thoroughly investigated before. Earlier reports indicated that the oxidative stress, caused by disrupted sleep patterns, is not uniformly spread throughout all the brain structures, with the hypothalamus being more severely affected [50]. Data show that REM disruption causes oxidative stress predominantly in the hippocampus [51] through the decrease in the antioxidant defenses [29, 52]. The results from our study clearly showed higher MDA concentration in all three brain structures, in the sleep-fragmented rats, while the activity of the antioxidant enzymes GPx and SOD was significantly decreased.

We have previously shown that sleep deprivation induces anxiety-linked behavior in both humans and animals. On the other hand, there is an association between anxiety and oxidative stress in the brain [25, 33, 53–55]. Present results indicated that there is a correlation between oxidative stress byproducts and anxiety behavior of the rats after sleep fragmentation. It seems that anxiety is especially associated with the increased oxidative changes in the hippocampus, while other brain structures are also affected. We herein demonstrated that lipid oxidative damage was present in all three explored brain structures, indicating notable oxidative stress as a result of sleep fragmentation. It seems that elevated SOD activity may protect cellular structures from oxidative damage [29, 56] which is in alignment with alleviated SOD activity, observed in our OSA model. When humans are exposed to sleep deprivation, they show a significant increase in oxidative stress biomarkers, most likely due to loss of plasma antioxidants [57, 58]. Consequently, OSA puts them at higher risk of developing a variety of neurological diseases [59] and neurocognitive impairment [60]. Having in mind the brain's modest antioxidant defenses, due to high lipid content, it is not a surprise that oxidative damage of lipids, proteins, and DNA molecules easily occurs [61, 62]. Moreover, oxidative stress could be implicated in the deleterious effect of sleep on other organ systems [63].

Contrary to the results that sleep disruption elevates oxidative stress in PV+ cells of the rat cerebral cortex by Harkness et al. [64], we did not observe significant alterations of hippocampal PV+ interneurons. Based on our results, an unaltered number of PV+ interneurons in CA1, CA2/3, and DG regions of the hippocampus in the SF group of rats (*vs*. TC and EC groups) could be reported. These differences could be attributed to the differences in experimental sleep modulation used and their duration. Therefore, our hypothesis on the reduction of PV+ interneurons upon SF could be rejected. Our hypothesis was based on the fact that the hippocampus, together with other circuits of the limbic system, amygdala, prefrontal cortex, and others is on the crossroad of fear, memory, and anxiety pathways and that PV+ interneuron inhibitory projects control anxiety [65, 66]. Loss of the hippocampal neurons coupled with anxiety-related behavior has been demonstrated in other studies [30, 67]. However, it should still be underlined that hippocampal PV+ interneurons [68] are involved in the behavioral patterns, including fear and anxiety [69], although they do not influence the mechanism of sleep fragmentation-evoked anxiety-like behavior.

Our results showed that the duration of sleep fragmentation is a significant factor of anxiety-linked behavior development, as well as that these effects are mediated via oxidative distress in the brain. Herein revealed, the sleep fragmentation-oxidative stress-anxiety axis contributes to our better understanding of pathophysiological processes, occurring due to disrupted sleep patterns.

## Data Availability

The datasets used and/or analyzed during the current study will be available from the corresponding author on reasonable request.

## Abbreviations

OSA: Obstructive sleep apnea
PV: Parvalbumin
TV: Treadmill control
EC: Motion, exercise control
SF: Sleep fragmentation
EPM: Elevated plus maze
OPF: Open field
LD: Light/dark
ROS: Reactive oxygen species
MDA: Malondialdehyde
GPx: Glutathione peroxidase
TBARS: Thiobarbituric acid reactive substances
SOD: Superoxide dismutase
P-SH: Protein thiol groups
DTNB: 5,5′-Dithiobis-(2-nitrobenzoic acid)
TNB: 5-Thio-2-nitrobenzoic acid
DG: Dentate gyrus
SE: Standard error
ATP: Adenosine triphosphate
REM: Rapid eye movement
SWS: Slow wave sleep
Nrf2: Nuclear factor erythroid 2-related factor 2.
